# Comparison of Peak Oxygen Uptake and Test-Retest Reliability of Physiological Parameters between Closed-End and Incremental Upper-Body Poling Tests

**DOI:** 10.3389/fphys.2017.00857

**Published:** 2017-10-30

**Authors:** Julia K. Baumgart, Knut Skovereng, Øyvind Sandbakk

**Affiliations:** Department of Neuromedicine and Movement Science, Centre for Elite Sports Research, Norwegian University of Science and Technology, Trondheim, Norway

**Keywords:** peak aerobic capacity, endurance performance, all-out, 3-min, exhaustion

## Abstract

**Objective:** To compare peak oxygen uptake (VO_2peak_) and the test-retest reliability of physiological parameters between a 1-min and a 3-min closed-end and an incremental open-end upper-body poling test.

**Methods:** On two separate test days, 24 healthy, upper-body trained men (age: 28.3 ± 9.3 years, body mass: 77.4 ± 8.9 kg, height: 182 ± 7 cm) performed a 1-min, a 3-min and an incremental test to volitional exhaustion in the same random order. Respiratory parameters, heart rate (HR), blood lactate concentration (BLa), rating of perceived exertion (RPE), and power output were measured. VO_2peak_ was determined as the single highest 30-s average. Relative reliability was assessed with the intra-class correlation coefficient (ICC_2, 1_) and absolute reliability with the standard error of measurement (SEM) and smallest detectable change (SDC).

**Results:** The incremental (3.50 ± 0.46 L·min^−1^ and 45.4 ± 5.5 mL·kg^−1^·min^−1^) and the 3-min test (3.42 ± 0.47 L·min^−1^ and 44.5 ± 5.5 mL·kg^−1^·min^−1^) resulted in significantly higher absolute and body-mass normalized VO_2peak_ compared to the 1-min test (3.13 ± 0.40 L·min^−1^ and 40.4 ± 5.0 mL·kg^−1^·min^−1^) (all comparisons, *p* < 0.001). Furthermore, the incremental test resulted in a significantly higher VO_2peak_ as compared to the 3-min test (*p* < 0.001). VO_2peak_ was significantly higher on day 1 than day 2 for the 1-min test (*p* < 0.05) and displayed a trend toward higher values on day 2 for the incremental test (*p* = 0.07). High and very high ICCs across all physiological parameters were found for the 1-min (0.827–0.956), the 3-min (0.916–0.949), and the incremental test (0.728–0.956). The SDC was consistently small for HR (1-min: 4%, 3-min: 4%, incremental: 3%), moderate for absolute and body-mass normalized VO_2peak_ (1-min: 5%, 3-min: 6%, incremental: 7%) and large for BLa (1-min: 20%, 3-min: 12%, incremental: 22%).

**Conclusions:** Whereas both the 3-min and the incremental test display high relative reliability, the incremental test induces slightly higher VO_2peak_. However, the 3-min test seems to be more stable with respect to day-to-day differences in VO_2peak_. The 1-min test would provide a reliable alternative when short test-duration is desirable, but is not recommended for testing VO_2peak_ due to the clearly lower values.

## Introduction

Exercise testing in a sitting position is relevant for determining upper-body physiological capacities and monitoring training progression in both Paralympic sitting athletes as well as able-bodied athletes involved in an upper-body sport. Various test protocols have been used to determine peak oxygen uptake (VO_2peak_) in upper-body modes, with the most common test procedure comprising incremental increases in workload until voluntary exhaustion (Bar-Or and Zwiren, 1975; Bhambhani et al., 1991; Leicht et al., 2009, 2013; Hutchinson et al., 2017). In addition, a 3-min self-paced closed-end test is a common procedure to assess VO_2peak_ in upper-body modes (Skovereng et al., 2013; Flueck et al., 2015; Hegge et al., 2015a,b; Baumgart and Sandbakk, 2016).

In cycling, the 3-min and incremental tests resulted in equally high VO_2peak_ values (Sperlich et al., 2011). The 3-min test additionally includes indices of performance and anaerobic capacity (i.e., accumulated oxygen deficit) (Losnegard et al., 2012), and therefore covers a more complementary set of measurements in a single test as compared to incremental workloads. In addition, the ability to increase the utilization of VO_2_ rapidly plays an important role in sports where high power outputs are produced over a relatively short time period. Examples are middle distance sports or intermittent activities such as cross-country skiing or Para cross-country skiing where hard work is performed in steep uphills followed by recovery in the subsequent downhill sections. Therefore, it would be of interest to explore the maximal rate of VO_2_ uptake during a test of shorter duration than traditionally employed. However, VO_2peak_ and corresponding physiological responses during closed-end tests of different duration and an incremental protocol have not yet been compared in upper-body exercise modes.

In an athletic context, sport-specificity of the testing mode is important in eliciting performance related peak responses (Roels et al., 2005). Upper-body poling is the most sports-specific mode for ice sledge hockey players and cross-country sit skiers as well as for testing upper-body capacity in cross-country skiers, biathletes and Nordic combined athletes. Thirty-nine Paralympic and 27 Olympic gold medals are contended for in these events, highlighting the importance of reliable test concepts for such sports. The 3-min and the incremental test are regarded reliable for the determination of VO_2peak_ as well as other physiological and perceptual parameters during arm-crank (Bar-Or and Zwiren, 1975; Leicht et al., 2009; Flueck et al., 2015) and wheelchair ergometry (Bhambhani et al., 1991; Leicht et al., 2013). However, the test-retest reliability of physiological parameters in upper-body poling needs to be established before meaningful differences between athletes and repeated tests within athletes can be interpreted.

The determination of test-retest reliability requires a relatively large group of homogeneous participants since most statistical measures of absolute and relative reliability are sensitive to population heterogeneity (Atkinson and Nevill, 1998; Hopkins, 2000; Weir, 2005). High test-retest reliability can solely be the result of a large spread of data points as compared to small intra-participant day-to-day variation (Atkinson and Nevill, 1998; Hopkins, 2000; Weir, 2005). Paralympic athletes represent a small group of participants with a large heterogeneity of physical capacities and are not preferable in this context.

Therefore, the aim of this study was to compare VO_2peak_ and the test-retest reliability between a 1-min and a 3-min closed-end and an incremental open-end upper-body poling test in able-bodied upper-body-trained participants. We hypothesized that the 3-min and the incremental upper-body poling tests would display high test-retest reliability as these protocols were previously found reliable for arm-crank and wheelchair ergometry. In line with previous research in cycling, we expected that the 3-min and incremental protocol would not differ in VO_2peak_.

## Materials and methods

### Participants

Twenty-four able-bodied upper-body-trained male individuals (age 28.3 ± 9.3, body mass 77.4 ± 8.9 kg, and height 1.8 ± 0.1 m) participated in this study. Participants were mainly cross-country skiers (*N* = 23) and additionally one rower who regularly trained cross-country skiing, all of whom participated in recreational or national level cross-country skiing and rowing races, respectively. All were highly trained with a running VO_2max_ of 66 ± 7 mL·kg^−1^·min^−1^ (range 53.0–75.9) and an average of 39 ± 11 (range 22.5–75) training hours per month (based on self-reported training hours from their training diary logs; www.olt-dagbok.net), most of which was endurance training and a considerable part employing the upper-body. The participants were instructed to refrain from heavy training and alcohol consumption 24 h before the start of the testing, caffeine intake the day of the testing and food intake 2 h before. A questionnaire was filled out on each day to monitor if the participants followed these instructions, as well as to exclude any prior illness or injury that might have interfered with the testing. The study was pre-approved by the Regional Committee for Medical and Health Research Ethics of Mid-Norway and conducted in accordance with the Declaration of Helsinki. All participants signed an informed consent form prior to participation in the experiment and were made aware that they could withdraw from the study at any point without providing an explanation.

### Overall design

The testing consisted of two test days, where participants performed a 1-min and a 3-min all out and an incremental test to exhaustion in an upper-body poling mode on a Concept2 ski-ergometer (Concept2, Inc., Morrisville, USA). Each participant performed the tests in the same order and at the same time of the day (to minimize the bias of diurnal variation in performance; Atkinson and Reilly, 1996). The test order was randomized between participants. The time between test days was a minimum of 48 h and an average of 4 ± 3 days (range 2–11 days). Before the start of the testing on the first day, the participants' body mass was assessed by the built-in weighing scale of a bioelectrical impedance analyzer (Inbody Co., Ltd., Seoul, Korea).

### Test set-up and familiarization

**Figure 1:**
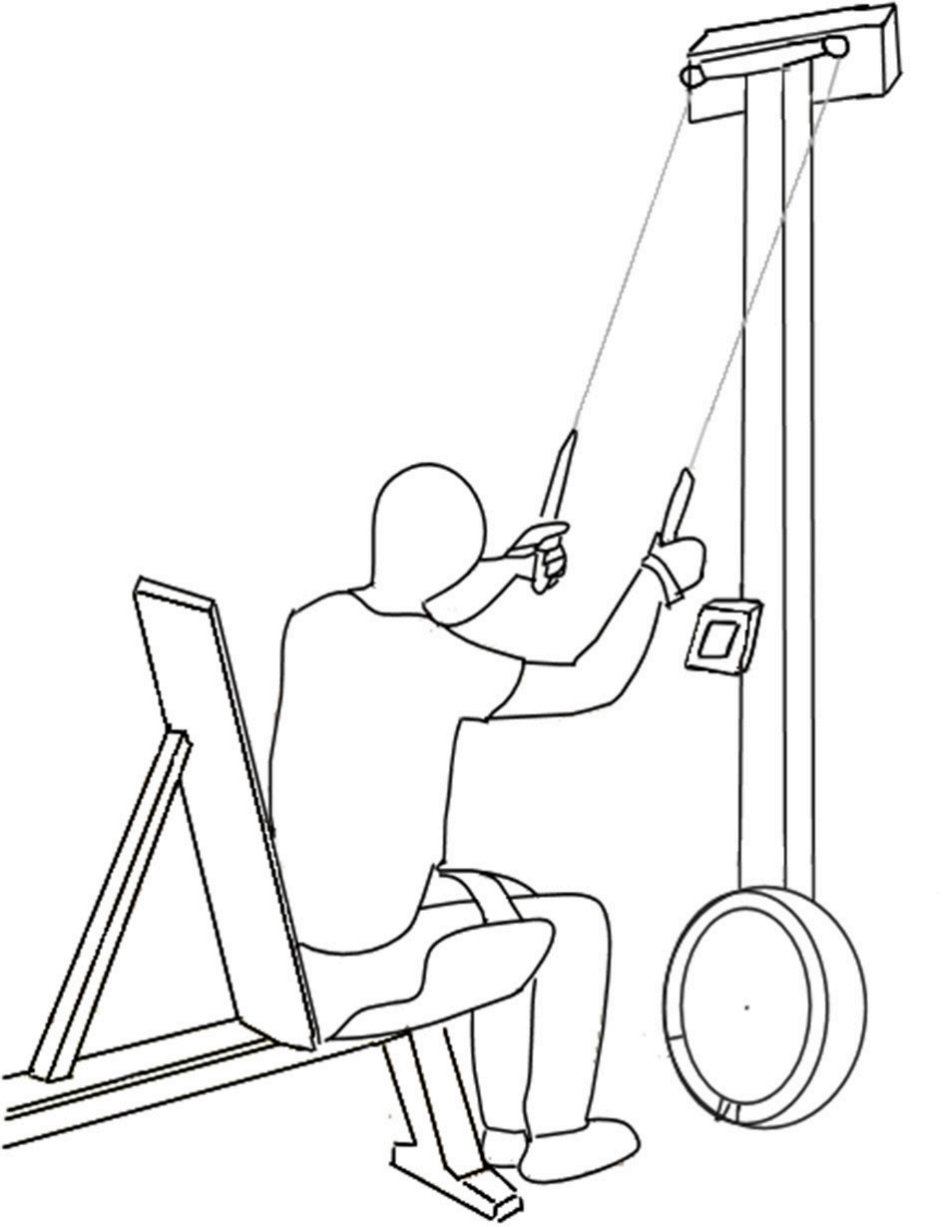
Test set-up with the participant in a sitting position, strapped around the hips and thighs, in front of the Concept2 ski-ergometer.

After being equipped with an oro-nasal mask (Hans Rudolph Inc, Kansas City, MO, USA) and a heart rate (HR) monitor (Polar Electro Inc., Port Washington, NY, USA), the participants tightly strapped themselves around the hips and thighs into a seat construction (see Figure 1). They were then familiarized with the test setup and mode by performing four times 5-min submaximal stages mode at an overall rate of perceived exertion of 9 (very light), 11 (light), 13 (somewhat hard), and 15 (hard) on a 6–20 Borg scale (Borg, 1982; Shephard et al., 1992).

### Test protocols

A 15-min break followed the submaximal familiarizing stages, before a standardized 5-min warm-up preceded each of the peak tests, consisting of three and two min at the power output of the third (RPE 13) and fourth submaximal stage (RPE 15), respectively. The third minute of warm-up was inter-dispersed by two 5-s sprints at 90–95% of maximal sprint power. Both, the 1-min and the 3-min test were self-paced closed-end tests with the instruction to find a power output that the participants thought they could maintain throughout the test. Three pacing strategies were possible: (1) a higher power output at the start with a drop toward the end (positive pacing), (2) a stable power output throughout the test (even pacing), and (3) a lower power output at the start with an increase toward the end (negative pacing) (Atkinson et al., 2007). Positive and negative pacing were defined as more than a 10% increase or decrease of the last 30-s average as compared to the initial 30-s average power output. The incremental test started at the individual power output of the third submaximal stage (rounded to the nearest 5-W value) and participants were instructed to continuously increase power output by 10 W every 30 s. Between each of the maximal tests, a rest period of 26 ± 3 min was given and the participants were optionally allowed to drink water or sports drink. It has previously been shown that a recovery period of 20 min between maximal tests allows participants to maintain performance (Weltman et al., 1979; Vesterinen et al., 2009; Moxnes and Moxnes, 2014).

VO_2_, VCO_2_, and VE were measured breath-by-breath using a spiroergometer (Oxycon Pro, Jaeger, Viasys BV, CA, USA) which was calibrated against a known mixture of gases (5% CO_2_, 15% O_2_) and a known air flow (from a 3 L syringe) prior to each test. HR was continuously recorded during the tests. A blood sample was taken 1 and 3 min after each test and blood lactate analyzed by a Biosen C-Line Sport lactate measurement system (EKF-diagnostic GmbH, Magdeburg, Germany). Overall (RPE_O_), respiratory (RPE_R_) and muscular rate of perceived exertion (RPE_M_), and were recorded after each test as described more in detail by Shephard et al. (1992). Power output per stroke was recorded by the skiergometer's internal software (Concept2, Morrisville, USA) and recorded by a Sony Alpha 58 video camera (Sony Corporation, NY, USA).

### Data processing and statistical analyses

A minimum number 21 participants was determined by a-priori analyses in G^*^Power 3.1, with an effect size of 0.65 (calculated as a Cohen's d based on VO_2peak_ values in a similar sample from Baumgart and Sandbakk, 2016, an alpha level of 0.05 and a power of 0.80. Two of the 24 participants were not able to complete the 1-min and the 3-min test on the second day. In these two tests, the data from 22 participants were analyzed. Breath-by-breath respiratory data was interpolated at individually fitted sample frequencies, resampled at 1-s intervals and 10, 30, and 60-s averages were calculated in MATLAB 8.1.0. (R2016a; Mathworks Inc., Natick, MA) The single highest 30-s average value was then identified as VO_2peak_ as recommended by Robergs et al. (2010) and used in the further analyses. The highest 10 and 60-s averages were used to investigate if changes in averaging procedure affected the results. Moving 3-s averages were calculated for the HR data and the highest value defined as peak heart rate (HR_peak_). The higher of the two blood lactate values was defined as peak blood lactate (BLa_peak_). Thirty seconds averages were calculated for the PO data and the highest value defined as peak power output (PO_peak_).

Data are presented as mean ± *SD* unless specified differently and an α level of 0.05 was employed to indicate statistical significance. All calculations and statistical tests were executed in Microsoft Excel (Version 2010, Microsoft Cooperation, The Microsoft Network, LLC, Richmond, USA) or in SPSS 22.0 (Software for Windows, SPSS Inc., Chicago, IL, USA).

#### Assumptions

The assumption of homoscedasticity was examined by plotting the individual test-retest differences against the individual means and by calculating the Pearson's *r* correlation coefficient between the two. A correlation of *r* > 0.25 was used to define heteroscedasticity (O'Donoghue, 2013). Heteroscedastic variables (VCO_2peak_, VE_peak_, and PO_peak_ of the 1-min test, and RPE_O_ and RPE_M_ of the incremental test) were transformed using the natural logarithm. However, this procedure did not improve the heteroscedasticity and we hence used the non-transformed data. The assumption of normally distributed test-retest differences was assessed by the Shapiro-Wilk test of normality and Normal Q-Q plots. Paired-samples *T*-Tests were used to assess systematic bias in physiological variables, RPE and PO_peak_ between the two test days. Independent-samples *T*-Tests were used to investigate whether using the same or unequal pacing strategies led to differences in the VO_2peak_ delta values from day 1 to day 2. A general linear mixed model was used to investigate the interaction effect of test order and the type of upper-body poling peak test on VO_2peak_.

#### Comparison of tests

Paired-samples *T*-tests were used to compare physiological variables, RPE_M_, RPE_R_, RPE_O_, and PO_peak_ between the three tests. To investigate the influence of a different averaging procedure on VO_2peak_ we compared 10 and 60-s average to the 30-s average described above with paired-samples *T*-tests. The average over day 1 and 2 for each variable was used for these comparisons.

#### Absolute reliability

Absolute reliability was assessed by the standard error of measurement (SEM) and the smallest detectable change (SDC). The SEM was calculated as *SD*_diff_/√2 (Hopkins, 2000), and the 80% SDC as SEM·1.28·√2 (Bland and Altman, 1986).

#### Relative reliability

Intraclass correlation coefficients (ICC_2, 1_) with 95% CI were calculated as a measure of relative reliability (Weir, 2005). Ranges of 0.26–0.49, 0.50–0.69, 0.70–0.89, and 0.90–1.0 were classified as low, moderate, high, and very high ICC according to Munro's criteria (Plichta et al., 2013).

## Results

### Comparison of tests

**Figure 2:**
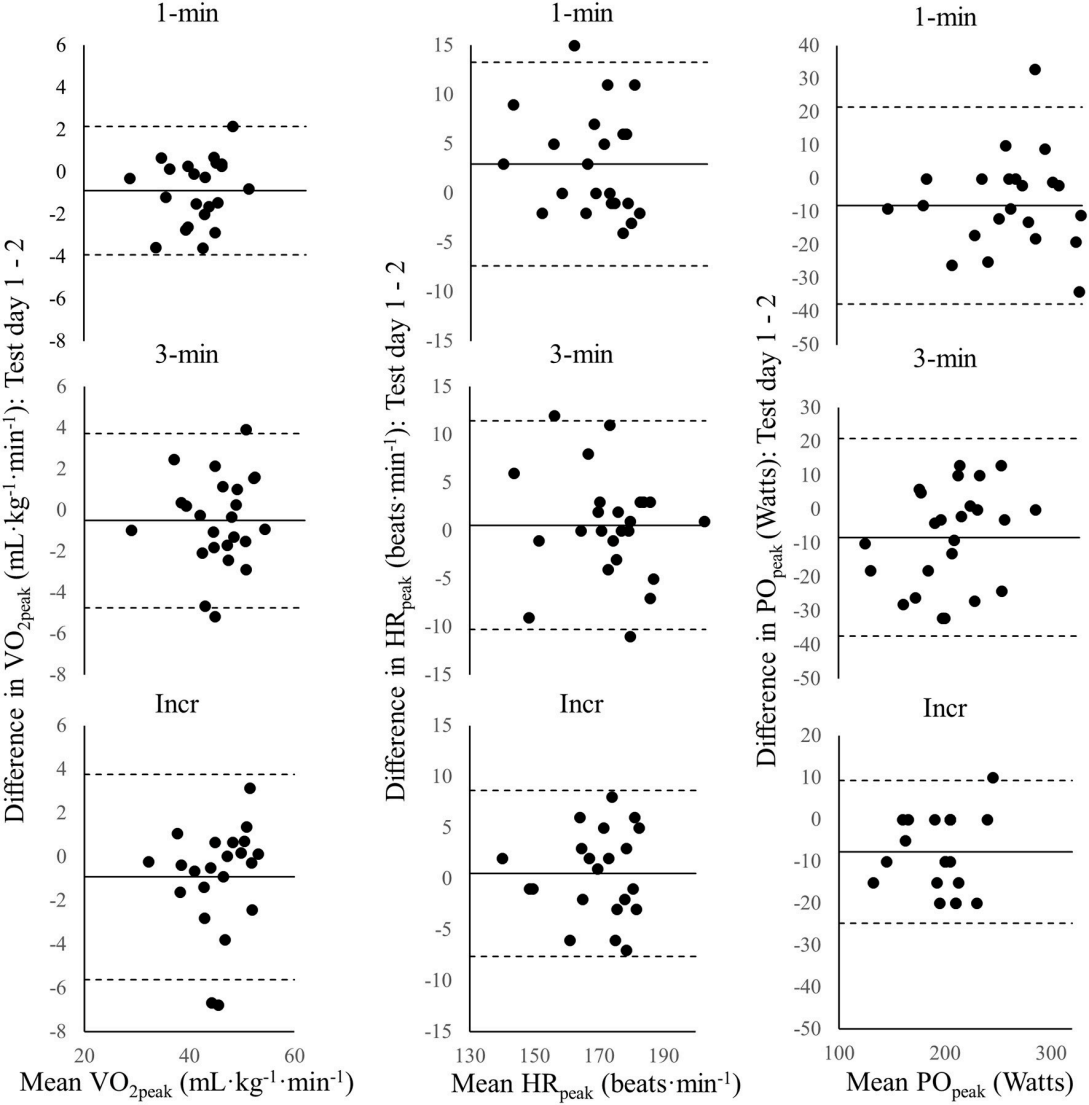
Bland-Altman plots for the individual mean body-mass normalized peak oxygen uptake (VO_2peak_), peak heart rate (HR_peak_), and peak power output (PO_peak_) of test day 1 and 2 vs. the difference between day 1 and 2 in VO_2peak_, HR_peak_, and PO_peak_ for the 1-min, the 3-min, and the incremental (Incr) test. The solid line is the group mean and the dotted lines indicate ±1.96·*SD*.

Individual differences and mean values of day 1 to day 2 and corresponding limits of agreement are visualized in Bland-Altman plots in Figure 2 and displayed in Table 1. All data used in the analyses of this study are found in Datasheet 1. Based on the average values of test day 1 and 2, the incremental (45.4 ± 5.5 mL·kg^−1^·min^−1^, 196 ± 28 W) and the 3-min test (44.5 ± 5.5 mL·kg^−1^·min^−1^, 201 ± 36 W) resulted in significantly higher VO_2peak_ and lower PO_peak_ as compared to the 1-min test (40.4 ± 5.0 mL·kg^−1^·min^−1^, 256 ± 47 W) (all *p* < 0.001). Additionally, the incremental test resulted in significantly higher VO_2peak_ (*p* < 0.03) (see Supplementary Figure 1). A plateau in VO_2peak_ (2 consecutive 30-s values within 2 mL·kg^−1^·min^−1^) was observed in ~80% of tests both during the 3-min and the incremental protocols of day 1 and 2, without any difference between test protocol or order of test day.

**Table 1:** Power output, physiological and perceptual parameters of test day 1 and 2 for a 1-min, a 3-min, and an incremental upper-body poling test in able-bodied, upper-body trained participants (means ± *SD*).

	**1-min**			**3-min**			**Incremental**		
	**Day 1**	**Day 2**	***p*-value**	**Day 1**	**Day 2**	***p*-value**	**Day 1**	**Day 2**	***p*-value**
Power output (Watt)	254 ± 46	259 ± 47^*^	<0.001	198 ± 40	203 ± 33^*^^,^^†^	<0.001	192 ± 29	200 ± 28^*^^,^^†^	<0.001
VO_2peak_ (mL·kg^−1^·min^−1^)	40.0 ± 5.2	40.9 ± 5.0^*^	0.014	44.2 ± 5.7	44.7 ± 5.5^†^	0.262	45.0 ± 5.8	45.9 ± 5.5^†^^,^^‡^	0.085
VO_2peak_ (L·min^−1^)	3.09 ± 0.42	3.17 ± 0.39^*^	0.007	3.40 ± 0.48	3.44 ± 0.46^†^	0.270	3.46 ± 0.45	3.54 ± 0.49^†^^,^^‡^	0.068
VCO_2peak_ (L·min^−1^)	3.46 ± 0.60	3.56 ± 0.49	0.152	4.03 ± 0.63	4.12 ± 0.62^†^	0.147	3.97 ± 0.52	4.19 ± 0.55^*^^,^^†^	0.001
VE (L·min^−1^)	145 ± 32	144 ± 27	0.677	161 ± 29	162 ± 30^†^	0.806	161 ± 23	165 ± 22^*^^,^^†^	0.044
HR_peak_ (beats·min^−1^)	168 ± 11	165 ± 12^*^	0.016	172 ± 13	171 ± 14^†^	0.611	171 ± 14	171 ± 14^†^	0.578
BLa_peak_ (mmol·L^−1^)	11.0 ± 2.1	10.9 ± 2.5	0.868	11.6 ± 2.4	11.8 ± 2.2^†^	0.489	11.4 ± 2.3	12.0 ± 2.2^†^	0.166
RPE_O_ (6–20)	18.1 ± 1.3	17.8 ± 1.4	0.318	18.1 ± 1.0	18.3 ± 1.2^†^	0.465	18.3 ± 0.9	18.3 ± 1.2	0.935
RPE_R_ (6–20)	17.6 ± 1.5	17.4 ± 1.6	0.554	17.7 ± 1.7	17.9 ± 1.8	0.484	17.7 ± 1.8	17.8 ± 1.7	0.544
RPE_M_ (6–20)	18.3 ± 1.1	18.2 ± 1.3	0.618	18.3 ± 1.1	18.4 ± 1.2	0.656	18.6 ± 0.9	18.4 ± 1.2	0.432

*Calculations are based on data from 22 participants for the 1-min and the incremental test and 24 participants for the 3-min test*.

*Peak oxygen uptake (VO_2peak_), peak carbon dioxide production (VCO_2peak_), minute ventilation (VE), peak heart rate (HR_peak_), peak blood lactate (BLa_peak_), overall rate of perceived exertion (RPE_O_), respiratory rate of perceived exertion (RPE_R_), muscular rate of perceived exertion (RPE_M_)*.

* *Significant differences from day 1 to day 2 at an alpha level of 0.05*.

† *Mean value of day 1 and day 2 significantly different from 1-min test mean value at an alpha level of 0.05*.

‡ Mean value of day 1 and day 2 significantly different from 3-min test mean value at an alpha level of 0.05

As compared to the 30-s average used in the above, employing a 10 or 60-s average would have resulted in significantly higher or lower VO_2peak_, respectively, in all three tests (all comparisons *p* < 0.001). When using 10-s averages, the difference in VO_2peak_ between the tests would have remained unchanged (1-min: 41.3 ± 5.3, 3-min: 45.4 ± 5.5, incremental: 46.3 ± 5.6 mL·kg^−1^·min^−1^) compared to using 30-s averages. However, using 60-s averages, the VO_2peak_ difference between the 1-min as compared to the 3-min and the incremental test would have increased, and the differences between the 3-min and the incremental test decreased (1-min: 32.6 ± 4.2, 3-min: 43.8 ± 5.7, incremental: 44.2 ± 5.4).

### Relative and absolute reliability

High and very high ICCs across all physiological outcome parameters and PO_peak_ were found for the 1-min, the 3-min and the incremental test (Table 2). In all three tests, the SDC was consistently small for HR_peak_ (1-min: 4%, 3-min: 4%, incremental: 3%), moderate for absolute and body-mass normalized VO_2peak_ (1-min: 5%, 3-min: 6%, incremental: 7%) as well as PO_peak_ (1-min: 10%, 3-min: 9%, incremental: 6%) and large for BLa_peak_ (1-min: 20%, 3-min: 12%, incremental: 22%).

**Table 2:** Interclass correlation coefficients (ICC) and [95% confidence interval (CI)], standard error of the measurement (SEM), smallest detectable change (SDC) of power output, physiological, and perceptual parameters for a 1-min, a 3-min and an incremental upper-body poling test in able-bodied, upper-body trained participants.

	**1-min**					**3-min**					**Incremental**				
	**ICC_2, 1_**	**95% CI**	**SEM**	**SDC**	**SDC%**	**ICC_2, 1_**	**95% CI**	**SEM**	**SDC**	**SDC%**	**ICC_2, 1_**	**95% CI**	**SEM**	**SDC**	**SDC%**
PO_peak_ (Watts)	0.946	[0.879–0.976]	10.7	19.4	7.6	0.923	[0.827–0.967]	10.5	19.0	9.4	0.955	[0.895-0.981]	6.1	11.1	5.7
VO_2peak_ (mL·kg^−1^·min^−1^)	0.956	[0.897–0.981]	1.1	2.0	4.9	0.942	[0.871–0.974]	1.5	2.8	6.2	0.933	[0.846-0.972]	1.7	3.1	6.7
VO_2peak_ (L·min^−1^)	0.952	[0.888–0.980]	0.1	0.2	5.4	0.949	[0.886–0.978]	0.1	0.2	6.2	0.938	[0.857–0.974]	0.1	0.2	6.9
VCO_2peak_ (L·min^−1^)	0.903	[0.781–0.959]	0.2	0.4	10.8	0.929	[0.843–0.969]	0.2	0.4	8.7	0.922	[0.822–0.967]	0.2	0.3	7.8
VE (L·min^−1^)	0.905	[0.786–0.959]	11.2	20.2	14.0	0.919	[0.822–0.964]	10.1	18.2	11.3	0.922	[0.822–0.967]	7.5	13.6	8.3
HR_peak_ (beats·min^−1^)	0.926	[0.831–0.969]	3.7	6.8	4.1	0.935	[0.856–0.971]	3.9	7.1	4.1	0.956	[0.897–0.981]	2.9	5.3	3.1
BLa_peak_ (mmol·L^−1^)	0.827	[0.629–0.924]	1.2	2.1	19.9	0.916	[0.816–0.963]	0.8	1.5	12.4	0.728	[0.450–0.877]	1.4	2.6	22.2
RPE_O_ (6–20)	0.707	[0.415–0.867]	0.9	1.6	8.9	0.833	[0.652–0.924]	0.6	1.1	5.8	0.429	[0.019–0.715]	0.9	1.7	9.1
RPE_R_ (6–20)	0.726	[0.447–0.876]	1.0	1.8	10.4	0.892	[0.767–0.952]	0.7	1.3	7.2	0.885	[0.744–0.951]	0.7	1.3	7.5
RPE_M_ (6–20)	0.580	[0.219–0.801]	0.9	1.6	8.9	0.791	[0.575–0.904]	0.6	1.2	6.3	0.679	[0.369–0.853]	0.8	1.4	7.4

*Calculations are based on data from 22 participants for the 1-min and the incremental test and 24 participants for the 3-min test*.

*Peak oxygen uptake (VO_2peak_), peak carbon dioxide production (VCO_2peak_), minute ventilation (VE), peak heart rate (HR_peak_), peak blood lactate (BLa_peak_), overall rate of perceived exertion (RPE_O_), respiratory rate of perceived exertion (RPE_R_), muscular rate of perceived exertion (RPE_M_), peak power output (PO_peak_)*.

Fourteen and 9 participants changed their pacing strategy from day 1 to day 2 for the 1-min and the 3-min test, respectively. However, there were no differences in VO_2peak_ between day 1 and day 2 for neither the 1-min (1.1 ± 1.7 vs. 0.8 ± 1.7 mL·kg^−1^·min^−1^, *p* = 0.67) nor the 3-min test (0.7 ± 2.0 vs. 0.2 ± 2.5 mL·kg^−1^·min^−1^, *p* = 0.53) when comparing those who changed and those who maintained a stable pacing strategy across test days (Supplementary Figures 2, 3).

There was no significant interaction between type of test and test order on VO_2peak_ (*p* = 0.779). Furthermore, VO_2peak_ did not differ between test day 1 and 2 for the 3-min test (below 1% change, *p* > 0.05) or the incremental test (~2%, *p* = 0.068 and 0.085), but increased significantly for the 1-min test (~2%, *p* = 0.014 and 0.007). PO_peak_ was significantly higher on test day 1 as compared to test day 2 for the 1-, the 3-min and the incremental test (2, 1, and 4%, all *p* < 0.015). In line with the increased PO_peak_, time to exhaustion significantly increased in the incremental test on day 2 (from 326 ± 63 to 346 ± 70 s, *p* = 0.003).

## Discussion

The aim of this study was to compare VO_2peak_ and test-retest reliability of physiological parameters between a 1-min and a 3-min closed-end and an incremental open-end upper-body poling test. The incremental and the 3-min test resulted in significantly higher VO_2peak_ as compared to the 1-min test, with the incremental test inducing slightly higher VO_2peak_ than the 3-min test. High and very high ICCs across all physiological parameters (0.728–0.956) and PO_peak_ (0.923–0.955) were found for all three tests. The SDC, as a measure of absolute reliability, was consistently small for HR_peak_, moderate for VO_2peak_ and PO_peak_, but large for BLa_peak_ for all three tests. Furthermore, the 3-min closed-end test was more stable with respect to day-to-day differences in VO_2peak_ as compared to the incremental and 1-min test.

We found that the 3-min and the incremental test resulted in higher VO_2peak_ values than the 1-min test, demonstrating that 1-min duration is too short for the kinetics of the cardio-respiratory system to respond to the increased work demand during upper-body work. This is supported by the absence of a plateau in VO_2peak_ during the 1-min test in all participants. In contrast, a plateau or drop in VO_2peak_ at the end of the 3-min and the incremental test was observed in the majority of our participants' tests. Even though no study had previously compared a 1-min test to a 3-min or incremental protocol, Price et al. (2014) found significantly lower VO_2peak_ during a 30-s Wingate test as compared to an incremental protocol. Furthermore, the incremental protocol led to slightly higher VO_2peak_ values than the 3-min test, which is in line with a comparable study in cross-country skiing (McGawley, 2017). However, the meaningfulness of the 1 mL·kg^−1^·min^−1^ higher VO_2peak_ during the incremental test in the current study can be questioned, since both tests reach a plateau and the difference was in part influenced by the averaging procedure. In the current study the highest 30-s average was chosen to indicate VO_2peak_ as recommended by Robergs et al. (2010). If we shortened the duration to the single highest 10-s VO_2peak_ value, the difference between tests would have stayed stable but the peak values were consistently higher. In contrast, if VO_2peak_ would have been defined over two consecutive 30-s periods, VO_2peak_ differences between the 3-min test and the incremental test become negligible, yet the difference in VO_2peak_ between both these tests and the 1-min test would have increased. The latter is logical since there is a lag in the VO_2_ kinetics response to the increased work demands included in the 1-min average. Thus, a 30-s average was deemed most appropriate in the current study to be able to compare tests, without taking the initial part of the test into consideration. Concluding from the above, the 1-min is not recommended as a VO_2peak_ test due to the clearly lower responses, whereas the 3-min test might slightly underestimate VO_2peak_, with the magnitude depending on the averaging procedure.

Our finding of high relative reliability of physiological parameters of the three upper-body poling tests, reflected by high ICCs, are in line with several previous studies. Three minutes closed-end and incremental arm crank ergometry tests displayed similar ICCs in not specifically upper-body trained able-bodied participants (Leicht et al., 2009; Flueck et al., 2015; Hutchinson et al., 2017) as well as incremental wheelchair ergometry or treadmill tests in athletes with different disabilities (Bhambhani et al., 1991; Leicht et al., 2013). The current data shows that the ranks of the participants remain stable from test day 1 to test day 2 also during upper-body poling. However, caution is needed in the ICC's interpretation as it is a measure of the between-subjects variation in relation to the within-subjects variation and can be inflated merely by sample heterogeneity (Atkinson and Nevill, 1998; Hopkins, 2000). In a previous study on the reliability of VO_2peak_ during an incremental wheelchair treadmill test, Leicht et al. (2013) tried to circumvent a too large spread between participants by grouping together athletes with similar disabilities and training status, which consequently lead to small group sample sizes. To achieve a sufficient sample size, yet at the same time have a homogeneous sample, we chose to recruit upper-body trained male participants for our study. Given that the participants in our study were highly and relatively similarly upper-body trained, we expected them to be more homogeneous than athletes with a disability. However, the coefficient of variation of the body-mass normalized VO_2peak_ of 12% during the incremental test was higher than the 8% variation found in a group of participants with lower-limb disabilities (Leicht et al., 2013). As such, the interpretability of the ICC as a measure of test-retest reliability in upper-body testing remains limited, since even homogeneous able-bodied participants show heterogeneous responses.

In comparison to relative reliability outcomes, absolute reliability measures provide the possibility to investigate the degree to which repeated measurements vary for individuals. In this study, the small SDC for HR_peak_ and moderate SDC for VO_2peak_ and PO_peak_ indicate acceptable absolute reliability of all three peak tests. However, the rather large SDCs for BLa_peak_, which are in line with previous studies (Leicht et al., 2009, 2013; Flueck et al., 2015), suggest that BLa_peak_ cannot be used as a reliable outcome measure in upper-body testing to exhaustion. That the SDC for absolute and body-mass normalized VO_2peak_ was only moderate can be explained by the higher values on day 2 for the 1-min and the incremental test, and for PO_peak_ for all three tests. The higher PO_peak_ and consequently higher VO_2peak_ values during the 1-min test on day 2 may be attributed to motivation to beat their previous score, although we have no data supporting this speculation. The higher VO_2peak_ during the incremental test on test day 2 can in part be explained by two participants having 0.5–0.6 L·min^−1^ and 7 mL·kg^−1^·min^−1^ higher absolute and body-mass normalized VO_2peak_, respectively. If the data of the two participants were excluded, VO_2peak_ differences between test day 1 vs. 2 would have become non-significant. During the incremental test, the higher PO_peak_ on day 2 is related to half of the participants being able to sustain at least one extra 30-s stage with a higher PO_peak_ on day 2. Overall, the 3-min test is the most stable with respect to day-to-day differences and, therefore, the most reliable of the three upper-body poling tests.

### Methodological considerations

Our participants were highly trained for the poling movement and the exercise intensities used in this study, and they familiarized themselves with four times 5-min submaximal warm-up stages. We, therefore, chose to not perform a separate familiarization session for the peak tests in advance. However, in hindsight and as a recommendation for further studies, a separate familiarization session should be performed for all tests if the main outcome measure is PO_peak_ and for the 1-min and the incremental test if the main outcome measure is VO_2peak_.

Furthermore, it remains to be investigated if other durations of the incremental test would result in different VO_2peak_ responses. As the participants in our study performed a thorough warm-up before starting the incremental test, we do not expect higher VO_2peak_ values with increases in duration of the test, but a follow-up study is needed to confirm this.

To be able to identify meaningful differences in body-mass normalized VO_2peak_ with paired comparison tests and similar participants in future studies, we estimated a sample size of 26 participants by *n* = 8·SDC^2^·(SEM^2^)^−1^ as proposed by Hopkins (2000). Relatively similar numbers apply for most of the variables used in our approach. It is often challenging to recruit so many similarly upper-body trained participants, and in particular when aiming to test Paralympic athletes which are homogeneous with respect to their disability. Therefore, as large sample sizes as possible should be aimed at, if necessary through international collaborations. In addition, detailed description of the testing procedure and individual data should be made available so high-quality meta-analyses can be performed in the future.

## Conclusion

In conclusion, we find acceptable absolute and relative reliability of a 1-min and a 3-min closed-end, and an incremental upper-body poling VO_2peak_ test in able-bodied, upper-body trained individuals. However, the 1-min test is not recommended as a VO_2peak_ test due to the clearly lower values than the 3-min and the incremental test. Whereas the 3-min test is more stable with respect to day-to-day differences in VO_2peak_, the incremental test leads to slightly higher VO_2peak_.

## Author contributions

JKB, KS, and ØS substantially contributed to the conception and design of the study. JKB and KS acquired the data. JKB analyzed the data and all three authors were involved in the interpretation of data. JKB drafted the study with KS and ØS critically revising it for important intellectual content. The final version sent in for publication was approved by all three authors. Agreement to be accountable for all aspects of the work was reached between JKB, KS, and ØS.

### Conflict of interest statement

The authors declare that the research was conducted in the absence of any commercial or financial relationships that could be construed as a potential conflict of interest.

## Supplementary material

Supplementary Figure 1 Bland-Altman plots for the individual mean body-mass normalized peak oxygen uptake (VO_2peak_) and peak power output (PO_peak_) of test day 1 and 2 vs. the difference in VO_2peak_ and PO_peak_ between the 3-min and the incremental test. The solid line is the group mean and the dotted lines indicate ±1.96·*SD*.

Supplementary Figure 2 Development of power output and VO_2_ (presented as 30-s averages) of the 1-min test plotted individually for each participant over time. Solid lines demark the power output, dotted lines the VO_2_. Black lines are for test day 1 and red lines for test day 2.

Supplementary Figure 3 Development of power output and VO_2_ (presented as 30-s averages) of the 3-min test plotted individually for each participant over time. Solid lines demark the power output, dotted lines the VO_2_. Black lines are for test day 1 and red lines for test day 2.

## Abbreviations

BLa_peak_: Peak blood lactate
HR_peak_: Peak heart rate
ICC: Interclass correlation coefficient
PO_peak_: Peak power output
RPE_M_: Muscular rate of perceived exertion
RPE_O_: Overall rate of perceived exertion
RPE_R_: Respiratory rate of perceived exertion
SDC: Smallest detectable change
SEM: Standard error of the measurement
VE: Minute ventilation
VO_2max_: Maximal oxygen uptake
VO_2peak_: Peak oxygen uptake
VCO_2peak_: Peak carbon dioxide production.
