# Corrigendum: Comparison of Peak Oxygen Uptake and Test-Retest Reliability of Physiological Parameters between Closed-End and Incremental Upper-Body Poling Tests

**DOI:** 10.3389/fphys.2018.00895

**Published:** 2018-07-03

**Authors:** Julia K. Baumgart, Knut Skovereng, Øyvind Sandbakk

**Affiliations:** Department of Neuromedicine and Movement Science, Centre for Elite Sports Research, Norwegian University of Science and Technology, Trondheim, Norway

**Keywords:** peak aerobic capacity, endurance performance, all-out, 3-min, exhaustion

In the original article, there was a mistake in Table 1 as published. The mistake concerns the peak power output values provided for the 1-min and the 3-min test. We initially based the calculations on the mean peak power output of the 1-min and the 3-min test on the values provided by the internal software of the Concept2 ski ergometer, which are cumulative averages (i.e., the first average is an average over the first 30 s, the second average is an average over the first minute, the third over one and a half minutes and so forth). However, when submitting this manuscript we recalculated the mean peak power output to reflect 30-s averages that are not cumulative and hence independent of the power output produced in the previous 30-s period.

**Table 1:** Power output, physiological and perceptual parameters of test day 1 and 2 for a 1-min, a 3-min and an incremental upper-body poling test in able-bodied, upper-body trained participants (means ± SD).

	**1-min**			**3-min**			**Incremental**		
	**Day 1**	**Day 2**	***p*-value**	**Day 1**	**Day 2**	***p*-value**	**Day 1**	**Day 2**	***p*-value**
Power output (Watt)	254 ± 46	259 ± 47^*^	< 0.001	198 ± 40	203 ± 33^*^, ^†^	< 0.001	192 ± 29	200 ± 28^*^, ^†^	< 0.001
VO_2peak_ (mL·kg^−1^·min^−1^)	40.0 ± 5.2	40.9 ± 5.0^*^	0.014	44.2 ± 5.7	44.7 ± 5.5^†^	0.262	45.0 ± 5.8	45.9 ± 5.5^†^,^‡^	0.085
VO_2peak_ (L·min^−1^)	3.09 ± 0.42	3.17 ± 0.39^*^	0.007	3.40 ± 0.48	3.44 ± 0.46^†^	0.270	3.46 ± 0.45	3.54 ± 0.49^†^,^‡^	0.068
VCO_2peak_ (L·min^−1^)	3.46 ± 0.60	3.56 ± 0.49	0.152	4.03 ± 0.63	4.12 ± 0.62^†^	0.147	3.97 ± 0.52	4.19 ± 0.55^*^, ^†^	0.001
VE (L·min^−1^)	145 ± 32	144 ± 27	0.677	161 ± 29	162 ± 30^†^	0.806	161 ± 23	165 ± 22^*^, ^†^	0.044
HR_peak_ (beats·min^−1^)	168 ± 11	165 ± 12^*^	0.016	172 ± 13	171 ± 14^†^	0.611	171 ± 14	171 ± 14^†^	0.578
BLa_peak_ (mmol·L^−1^)	11.0 ± 2.1	10.9 ± 2.5	0.868	11.6 ± 2.4	11.8 ± 2.2^†^	0.489	11.4 ± 2.3	12.0 ± 2.2^†^	0.166
RPE_O_ (6–20)	18.1 ± 1.3	17.8 ± 1.4	0.318	18.1 ± 1.0	18.3 ± 1.2^†^	0.465	18.3 ± 0.9	18.3 ± 1.2	0.935
RPE_R_ (6–20)	17.6 ± 1.5	17.4 ± 1.6	0.554	17.7 ± 1.7	17.9 ± 1.8	0.484	17.7 ± 1.8	17.8 ± 1.7	0.544
RPE_M_ (6–20)	18.3 ± 1.1	18.2 ± 1.3	0.618	18.3 ± 1.1	18.4 ± 1.2	0.656	18.6 ± 0.9	18.4 ± 1.2	0.432

*Calculations are based on data from 22 participants for the 1-min and the incremental test and 24 participants for the 3-min test*.

Peak oxygen uptake (VO_2peak_), peak carbon dioxide production (VCO_2peak_), minute ventilation (VE), peak heart rate (HR_peak_), peak blood lactate (BLa_peak_), overall rate of perceived exertion (RPE_O_), respiratory rate of perceived exertion (RPE_R_), muscular rate of perceived exertion (RPE_M_).

* *Significant differences from day 1 to day 2 at an alpha level of 0.05*.

† *Mean value of day 1 and day 2 significantly different from 1-min test mean value at an alpha level of 0.05*.

‡ *Mean value of day 1 and day 2 significantly different from 3-min test mean value at an alpha level of 0.05*.

The corrected Table 1 appears below.

In the original article, there was an error. The mistake is in line with what is described in the above.

A correction has been made to the section **Results**, subsection Comparison of Tests, paragraph 1:

Based on the average values of test day 1 and 2, the incremental (45.4 ± 5.5 mL·kg^−1^·min^−1^, 196 ± 28 W) and the 3-min test (44.5 ± 5.5 mL·kg^−1^·min^−1^, 201 ± 36 W) resulted in significantly higher VO_2peak_ and lower PO_peak_ as compared to the 1-min test (40.4 ± 5.0 mL·kg^−1^·min^−1^, 256 ± 47 W) (all *p* < 0.001). Additionally, the incremental test resulted in significantly higher VO_2peak_ (*p* = 0.03).

The authors apologize for these errors and state that this does not change the scientific conclusions of the article in any way.

The original article has been updated.

## Conflict of interest statement

The authors declare that the research was conducted in the absence of any commercial or financial relationships that could be construed as a potential conflict of interest.

