# Supplementary figures and images for: Comparison of Peak Oxygen Uptake and Test-Retest Reliability of Physiological Parameters between Closed-End and Incremental Upper-Body Poling Tests
Source: Front Physiol. 2017 Oct 30;8:857. doi: 10.3389/fphys.2017.00857 (PMC5670144; doi:10.3389/fphys.2017.00857)

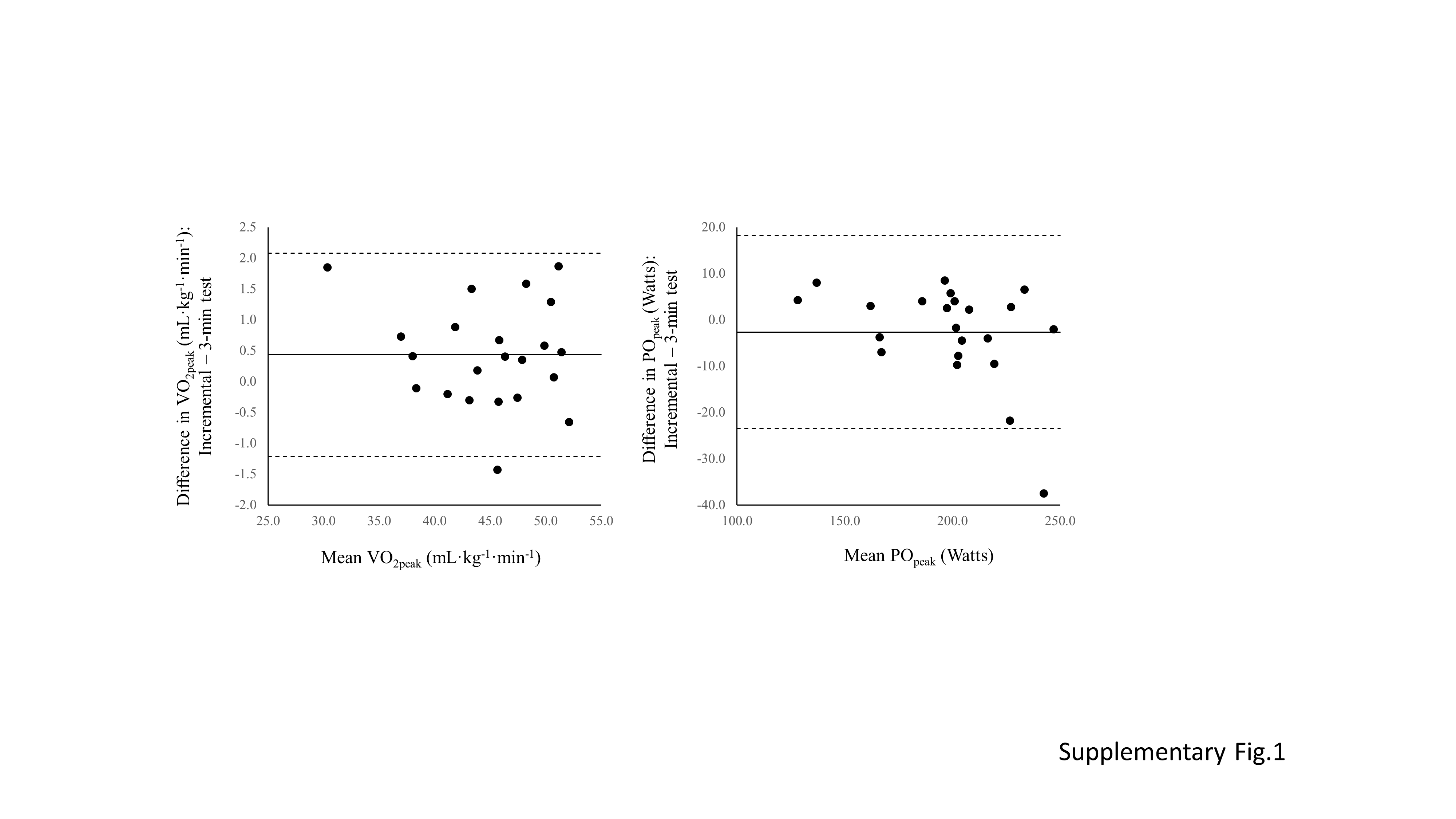

Supplement: Supplementary Figure 1 — Bland-Altman plots for the individual mean body-mass normalized peak oxygen uptake (VO2peak) and peak power output (POpeak) of test day 1 and 2 vs. the difference in VO2peak and POpeak between the 3-min and the incremental test. The solid line is the group mean and the dotted lines indicate ±1.96·SD. [file Image1.tif]

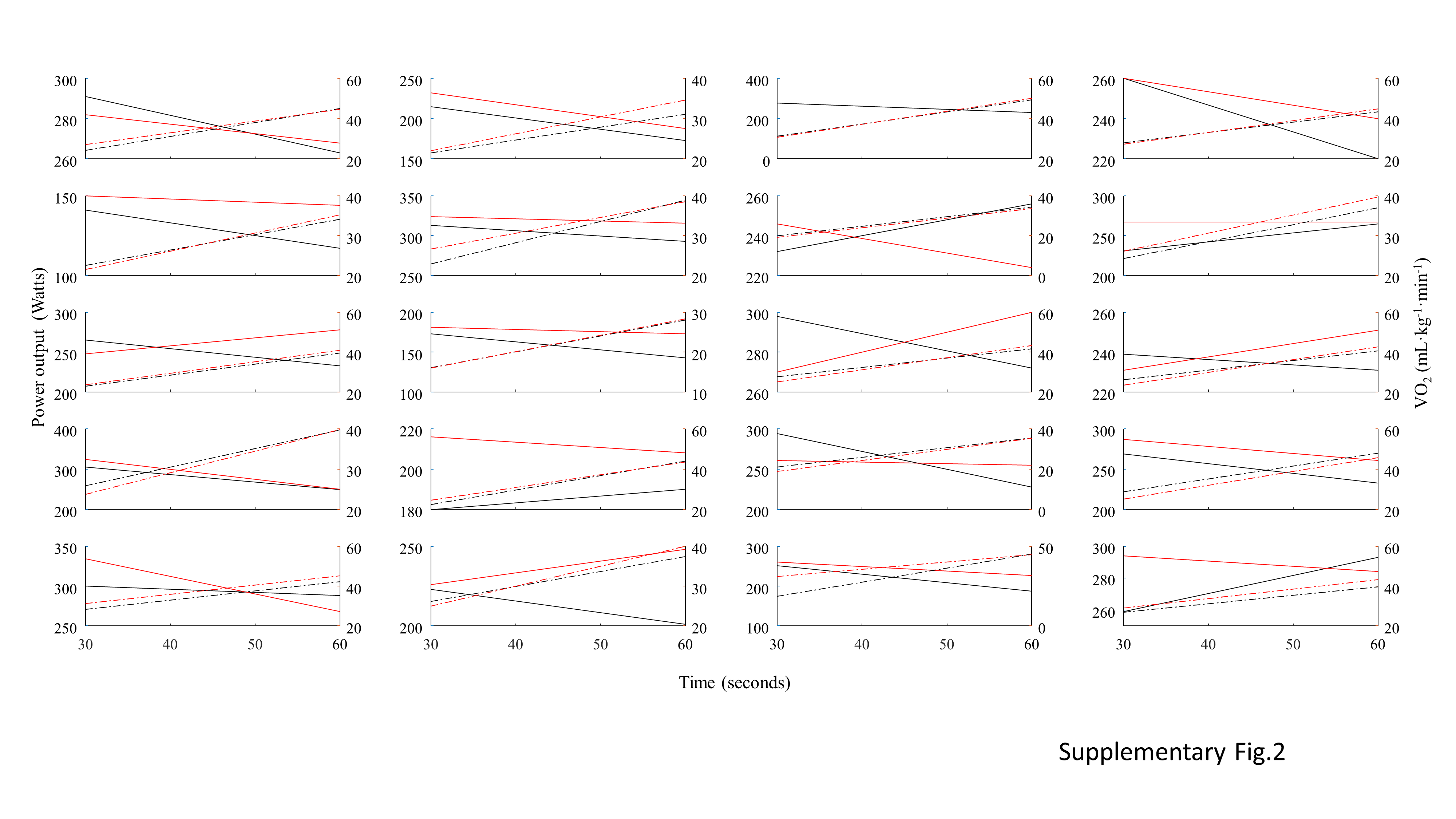

Supplement: Supplementary Figure 2 — Development of power output and VO2 (presented as 30-s averages) of the 1-min test plotted individually for each participant over time. Solid lines demark the power output, dotted lines the VO2. Black lines are for test day 1 and red lines for test day 2. [file Image2.tif]

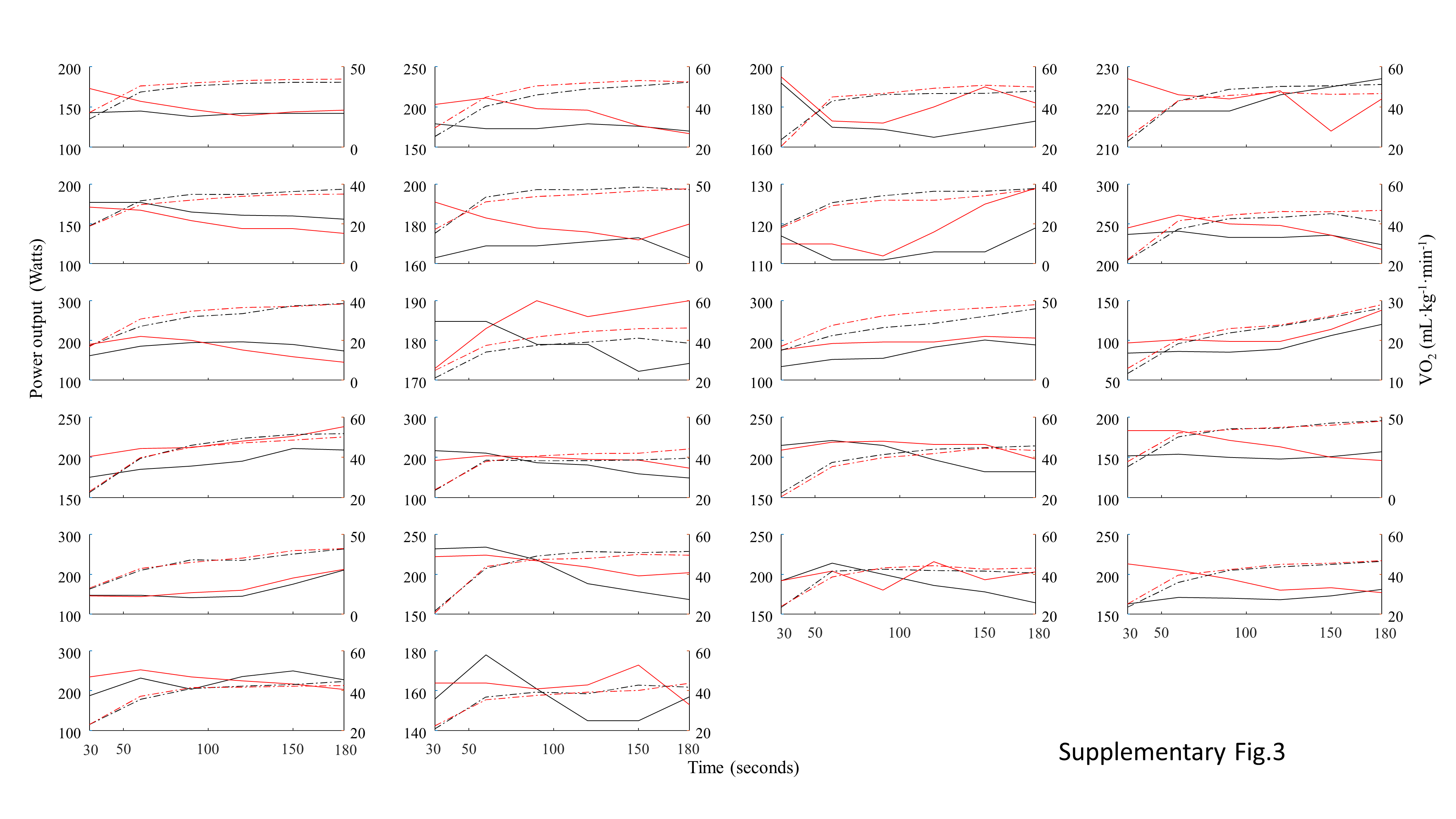

Supplement: Supplementary Figure 3 — Development of power output and VO2 (presented as 30-s averages) of the 3-min test plotted individually for each participant over time. Solid lines demark the power output, dotted lines the VO2. Black lines are for test day 1 and red lines for test day 2. [file Image3.tif]
